# Multicenter external validation of the radical cystectomy pentafecta in a European cohort of patients undergoing robot-assisted radical cystectomy with intracorporeal urinary diversion for bladder cancer

**DOI:** 10.1007/s00345-021-03753-y

**Published:** 2021-07-03

**Authors:** P. Baron, Z. Khene, F. Lannes, G. Pignot, A. S. Bajeot, G. Ploussard, G. Verhoest, A. Gasmi, O. Perrot, M. Roumiguie, K. Mori, G. E. Cacciamani, M. Rouprêt, F. Bruyère, B. Pradere

**Affiliations:** 1 Department of Urology, University Hospital of Tours, Loire Valley, Tours, France; 2 Department of Urology, Rennes University Hospital, Rennes, France; 3 Service de Chirurgie Oncologique 2, Institut Paoli-Calmettes, Marseille, France; 4 Department of Urology, Andrology and Renal Transplantation, CHU Rangueil, Toulouse, France; 5 Department of Urology, Clinique La Croix du Sud, Toulouse, France; 6 Department of Urology, Sorbonne Université, GRC no 5, predictive onco-urology, AP-HP, Hôpital Pitié-Salpêtrière, Urology, 75013 Paris, France; 7 Department of Urology, The Jikei University School of Medicine, Tokyo, Japan; 8 USC Institute of Urology, University of Southern California, Los Angeles, CA USA; 9 Department of Urology, Comprehensive Cancer Center, Medical University of Vienna, Vienna, Austria

**Keywords:** Robotics, Bladder neoplas, Cystectomy, Urinary diversion, Urothelial carcinoma, Validation

## Abstract

**Objective:**

To perform an external validation of this RC-pentafecta.

**Method:**

Between January 2014 and December 2019, 104 consecutive patients who underwent RARC with ICUD within 6 urological centers were analyzed retrospectively. Patients who simultaneously demonstrated negative soft tissue surgical margins (STSMs), a lymph node (LN) yield ≥ 16, absence of major (Clavien–Dindo grade III–V) 90-day postoperative complications, absence of UD-related long-term sequelae, and absence of 12-month clinical recurrence were considered to have achieved RC-pentafecta. A multivariable logistic regression model was used to measure predictors for achieving RC-pentafecta. We analyzed the influence of this RC-pentafecta on survival, and the impact ofthe surgical experience.

**Results:**

Since 2014, 104 patients who had completed at least 12 months of follow-up were included. Over a mean follow-up of 18 months, a LN yield ≥ 16, negative STSMs, absence of major complications at 90 days, and absence of UD-related surgical sequelae and clinical recurrence at ≤ 12 months were observed in 56%, 96%, 85%, 81%, and 91% of patients, respectively, resulting in a RC-pentafecta rate of 39.4%. Multivariate analysis showed that age was an independent predictor of pentafecta achievement (odds ratio [OR], 0.96; 95% confidence interval [CI], 0.90. 0.99; *p* = 0.04). The surgeon experience had an impact on the validation of the criteria.

**Conclusion:**

This study confirmed that the RC-pentafecta is reproducible and could be externally used for the outcome assessment after RARC with ICUD. Therefore, the RC-pentafecta could be a useful tool to assess surgical success and its impact on different outcomes.

## Introduction

Every year, 2.7 million people worldwide are diagnosed or treated for bladder cancer (BCa) [1, 2]. For locally advanced muscle-invasive bladder cancer (MIBC) without evidence of metastasis, neoadjuvant platinum-based systemic therapy and radical cystectomy (RC) with extensive bilateral pelvic lymph node dissection (PLND) is the recommended first-line curative treatment [3]. However, this procedure is technically demanding and has a complication rate of approximately 25% with a mortality rate of 1–2% regardless of the surgical approach [4]. Several techniques, including the robot-assisted approach, have been developed to reduce the perioperative morbidity and mortality rates.

The use of composite criteria to assess the quality of surgical procedures is being increasingly described in the literature. A trifecta developed by Brasseti et al. evaluate robot-assisted radical cystectomy on surgical, oncological and functional criteria but has been developed only for intracorporeal orthotopic neobladder procedure [5]*.* A RC-pentafecta was proposed in 2015 [6]; where the surgical approach of the total cohort was open in 98.7% of cases. This RC-pentafecta was modified by Cacciamani et al. in 2020 [7] to combine functional and oncological criteria for the evaluation of RARC with intracorporeal urinary diversion (ICUD). Although these criteria are relevant for assessing the learning curve and the quality of surgery, an external validation remains still required before a wide acceptance and use in future studies.

The objective of our study was to carry out an external validation of RC-pentafecta by evaluating the perioperative and oncological results of RARC-ICUD.

### Patients and methods

This is a multicenter retrospective study performed between January 2014 and December 2019 at six urological centers. All consecutive patients who had a RARC-ICUD for muscle-invasive bladder cancer or high-risk no muscle-invasive bladder cancer, whatever pathology, were included. Only patients with a follow-up period of at least 12 months were included. Patients who had undergone open or pure laparoscopic surgery and those with ECUD (*n* = 99) were excluded. Patients with metastasis at the time of surgery were also excluded.

Robot-assisted surgery was performed using a Da Vinci ® Surgical System Si or Xi robot (Intuitive Surgical Inc., Sunnyvale, CA, USA) using four arms according to the surgical technique conventionally described and according to the surgeon experience [8]. The decision to perform an orthotopic neobladder or ileal conduit was at the discretion of the surgeon according to the preoperative assessment and the patient- and disease-related features [3].

### Perioperative evaluation

For each patient, preoperative characteristics were collected, including age, sex, body mass index (BMI), American Society of Anesthesiology (ASA) score, Eastern Cooperative Oncology Group (ECOG) scale, cardiovascular co-morbidities (hypertension, diabetes, smoking), preoperative creatinine and hemoglobin, and the use and regimen of neoadjuvant chemotherapy (NAC). The collected perioperative variables included the type of urinary diversion, operating time (min), estimated blood loss (EBL) (quantified by the sum of suction device and pads) and perioperative transfusions. The postoperative data included: complications within 90 days according to the Clavien–Dindo (CD) classification [9] by separating minor (CD ≤ II) and major (CD ≥ III) postoperative complications, urinary diversion (UD)-related sequelae as previously reported [10] within 12 months, length of hospital stay (LOS), and the 90 days readmission rate. UD-related sequelae complications were defined by all urinary events within 12 months after surgery, and included urinary fistulas, uretero-ileal anastomotic strictures, stoma hernias, and stones [10, 11].

Oncological results were assessed for the overall recurrence rate, with recurrences within 12 months divided into local and distant recurrences. All recurrences were collected after the last imaging assessment. Death due to any cause and specific cause death were reported according to the patient's death certificate or medical record.

### Anatomopathological evaluation

All excised tissues were evaluated according to the standard anatomopathological protocol of each center and the staging system of the American Joint Committee on Cancer [12]. The tumor grade was determined according to the 2016 World Health Organization (WHO) classification [13]. Positive margins were defined by the presence of tumor cells facing the section slice. The following results were recorded: the numbers of lymph nodes removed, the anatomopathological T and N stage, and the rate of patients with locally advanced disease defined by a pT ≥ 3 stage and/or a positive N stage.

### Patient follow-up

The patients were monitored according to the protocol of each center and the recommendations of the European Association of Urology (EAU) [3, 14].

### External validation of the pentafecta

To be considered a validated pentafecta, patients had to meet all of the following five criteria: removal of $\ge$ 16 lymph nodes, negative soft tissue surgical margins, absence of major complications (CD ≥ III) within 90 days, absence of UD-related sequelae within 12 months as previously defined [10] and absence of local or distant recurrence within 12 months.

To assess the learning curve influence on the pentafecta validation, the surgeon’s experience was classified according to three stages (the cut-off between groups was obtain by consensus between all the experts among co-authors): unexperienced surgeon (< 10 RARC), intermediate (10–30 RARC) and experienced surgeon (> 30 RARC with intracorporeal or extracorporeal urinary diversion).

### Statistical analysis

The Chi-squared and Fisher exact tests were used to compare qualitative variables, and Welch's test was used to compare quantitative variables. Continuous variables were expressed as means and medians with standard deviations (SD) and interquartile range (IQR). After selection of predictors in unvariable, multivariable analysis was performed by logistic regression to identify predictors of "validated RC-pentafecta". The data set with a *p* value < 0.2 in univariable analysis was studied in multivariable analysis. The overall and recurrence-free survival was analyzed using the Kaplan–Meier method, and the log-rank test was applied to test the difference between the two groups.

All reported *p* values were two-sided, and statistical significance was set at < 0.05. All statistical analyses were performed using R version 3.6.3.

## Results

### Patient characteristics

One hundred and four patients who underwent RARC-ICUD were included. Within the cohort, the mean age of the patients was 65.8 (± 9.63) years, and NAC was administered in 68.2% (*n* = 71) of cases. The patient characteristics are summarized in Table 1*.*

**Table 1:** Patient’s characteristics and perioperative outcomes

Characteristics and perioperative outcomes	Total	RC-pentafecta validated	RC-pentafecta not validated	P*
Patients *n* (%)	104	41 (39.4%)	61 (60.6%)	
Age, years				
Mean (SD)	65.8 (± 9.63)	63.4 (± 9.64)	67.5 (± 8.98)	**0.03**
BMI, kg/m^2^				
Mean (SD)	26.1 (± 4.93)	25.9 (± 3.59)	26.3 (± 5.75)	0.67
Gender *n* (%)				0.55
Male	88 (84.6%)	36 (87.8%)	52 (85.2%)	
Female	16 (15.4%)	5 (12.2%)	9 (14.8%)	
ASA score *n* (%)				
ASA ≤ II	86 (82.6%)	37 (90.2%)	47 (77.0%)	0.09
ASA > II	18 (17.4%)	4 (9.8%)	14 (33.0%)	
Diabetes *n* (%)	11 (10.6%)	0 (0%)	11 (18.0%)	** < 0.01**
ECOG *n* (%)				
0	71 (68.2%)	26 (63.4%)	43 (70.4%)	1
1	31 (29.8%)	14 (34.1%)	17 (27.8%)	
2	2 (2.0%)	1 (2.5%)	1 (1.8%)	
NAC *n* (%)	71 (68.2%)	29 (70.7%)	42 (68.9%)	0.84
Clinical T-stage *n* (%)				
cT ≤ 2				
cTIS	2 (1.9%)	0 (0%)	1 (0%)	0.96
cTa	6 (5.8%)	1 (2.4%)	5 (8.4%)	
cT1	13 (12.5%)	4 (9.8%)	8 (13.3%)	
cT2	79 (76.0%)	33 (80.5%)	46 (76.5%)	
cT ≥ 3				
cT3	3 (2.9%)	2 (4.9%)	1 (1.8%)	
cT4	1 (0.9%)	1 (2.4%)	0 (0%)	
Clinical N-stage *n* (%)				
cN0	93 (89.4%)	39 (95.1%)	52 (85.2%)	0.19
cN1	11 (10.6%)	2 (4.9%)	9 (14.8%)	
cN2	0 (0%)	0 (0%)	0 (0%)	
cN3	0 (0%)	0 (0%)	0 (0%)	
cNx	0 (0%)	0 (0%)	0 (0%)	
Operative time, min				
Mean (SD)	366 (± 94.2)	359 (± 80.8)	376 (± 99.1)	0.34
Median (IQR)	360 [300; 436]			
EBLs, ml				
Mean (SD)	411 (± 281)	450 (± 321)	396 (± 247)	0.37
Median (IQR)	320 [234; 500]			
Type of UD *n* (%)				
Incontinent	29 (27.8%)	8 (19.5%)	19 (31.1%)	0.19
Continent	75 (72.2%)	33 (80.5%)	42 (68.9%)	
Transfusions *n* (%)				
Perioperative	2 (2.8%)	1 (2.4%)	1 (1.6%)	1
Postoperative	11 (10.6%)	4 (9.8%)	7 (11.4%)	1
Overall 90 day complications *n* (%)				
Overall	62 (59.6%)	22 (53.7%)	40 (62.5%)	0.06
Minor	46 (75.8%)	22 (100%)	24 (60.0%)	0.15
Major^b^	16 (24.2%)	0 (0%)	16 (40.0%)	** < 0.001**
Overall 12 month UD-related surgical sequelae^a^ *n* (%)	20 (19.2%)	0 (0%)	20 (32.8%)	** < 0.001**
LOS, days				
Mean (SD)	13.7 (± 7.47)	13.0 (± 3.92)	14.2 (± 9.07)	0.38
90 day readmission rate *n* (%)	29 (27.9%)	7 (17.1%)	22 (36.1%)	**0.04**
Adjuvant Chemotherapy *n* (%)	4 (3.8%)	1 (2.4%)	3 (4.9%)	1
ERAS protocol *n* (%)	53 (50.9%)	19 (46.3%)	32 (52.5%)	0.54

Bold values mean statistically significant

^a^Test: Welch, Fisher and Chi2

^b^Key outcomes included in the RC-Pentafecta

RC, radical cystectomy; ASA, American society of anesthesiologists; BMI, body mass index; ECOG, Eastern Cooperative Oncology Group; NAC, neoadjuvante chemotherapy; EBL, estimated blood loss; UD, urinary diversion; LOS, length of stay

### Perioperative outcomes

The perioperative results are summarized in Table 1. The mean operating time (SD) was 366 (± 94.2) min, and the mean EBL was 411 ml (± 281). Urinary diversion by ileal conduit was performed in 27.8% of cases (*n* = 29), and orthotopic neobladder was performed in 72.2% (*n* = 75) of cases. The mean LOS was 13.7 (± 7.47) days, with a 90-day readmission rate of 27%. The median LOS was significantly longer for the patient with neobladder in comparison to ileal conduit (13.0 [10.2; 15.8] vs 11.0 [8.0; 13.2]; *p* = 0,017).

The overall complication rate was 59.6% (*n* = 62), including 25.8% (*n* = 16) major complications (CD ≥ III). The rate of urinary complications in the first 12 months was 19.2%, among them, more than two-thirds (70.3%) were uretero-ileal strictures*.*

### Pathological outcomes

A complete pathological response on specimen (pT0) patients constituted 37.5% (*n* = 39) of the total cohort, 74.4% of them had received NAC (ypT0). The mean number of lymph nodes yield was 17.9 (± 7.89), 29 (27.9%) patients presented with locally advanced disease (pT ≥ 3 and/or N +), and 4 had a positive margin (3.8%). All the results are reported in Table 2.

**Table 2:** Pathological and oncological outcomes

Pathological and oncological outcomes	Total	RC-pentafecta validated	RC-pentafecta not validated	*P*^a^
Patients *n* (%)	104	41 (39.4%)	61 (60. 6%)	
LN count				
Mean (DS)	17.9 (± 7.89)	22.7 (± 6.41)	14.6 (± 7.07)	** < 0.001**
Median (IQR)	17.0 [12.0; 22.0]			
LN count ≥ 16^b^ *n* (%)	57 (54.8%)	41 (100%)	16 (26.2%)	** < 0.001**
PSM^b^ *n* (%)	4 (3.8%)	0 (0%)	4 (6.6%)	0.29
Pathological T-stage *n* (%)				
pT ≤ 2				
pT0	39 (37.5%)	20 (48.8%)	18 (29.5%)	0.50
pTis	17 (16.3%)	7 (17.1%)	10 (16.4%)	
pT1	12 (11.5%)	5 (12.2%)	6 (9.8%)	
pT2	14 (13.5%)	3 (7.3%)	11 (18.1%)	
pT ≥ 3				
pT3	21 (20.2%)	6 (14.6%)	15 (24.6%)	
pT4	1 (1.0%)	0 (0%)	1 (1.6%)	
Pathological N-stage *n* (%)				
pN0	86 (83.7%)	38 (92.7%)	47 (77.1%)	0.23
pN1	8 (7.7%)	3 (7.3%)	5 (8.2%)	
pN2	6 (5.8%)	0 (0%)	6 (9.8%)	
pN3	1 (1.0%)	0 (0%)	1 (1.6%)	
pNx	2 (1.8%)	0 (0%)	2 (3.3%)	
None organ confined *n* (%)	29 (27.9%)	7 (17.1%)	22 (36.1%)	**0.04**
Follow-up				
Mean (DS)	23.5 (± 15.6)	23.0 (± 13.3)	24.0 (± 17.3)	0.75
Median (IQR)	18.0 [12.8; 29.0]			
Overall recurrence rate *n* (%)	16 (15.4%)	2 (4.9%)	14 (22.9%)	**0.02**
Overall recurrence rate *n* (%)				
≤ 12 month^b^	7 (6.7%)	0 (0%)	7 (16%)	0.08
Local	1 (14.3%)	0 (0%)	1 (14.3%)	0.09
Distant	5 (71.4%)	0 (0%)	5 (71.4%)	
Both	1 (14.3%)	0 (0%)	1 (14.3%)	
Overall death *n* (%)	4 (3.9%)	2 (4.9%)	2 (3.3%)	1
Specific death *n* (%)	2 (1.9%)	1 (2.4%)	1 (1.6%)	1

Bold values mean statistically significant

^a^Test: Welch, Chi2 and Fisher

^b^Key outcomes included in the RC-Pentafecta

RC, radical cystectomy; LN, lymph node; PSM, positive surgical margin; none organ confined: pT ≥ 3 et /ou N +

### Oncological outcomes

With a median follow-up period of 18 months, 16 patients (15.4%) experienced recurrence during the 12 months postoperatively; among them, 14.3% presented with local recurrence, while 71.4% had distant recurrence (Table 2). The overall mortality rate was 3.9% (*n* = 4), and the specific death rate was 1.9% (*n* = 2).

### External validation of the RC-pentafecta and the learning curve impact

**Fig. 1:**
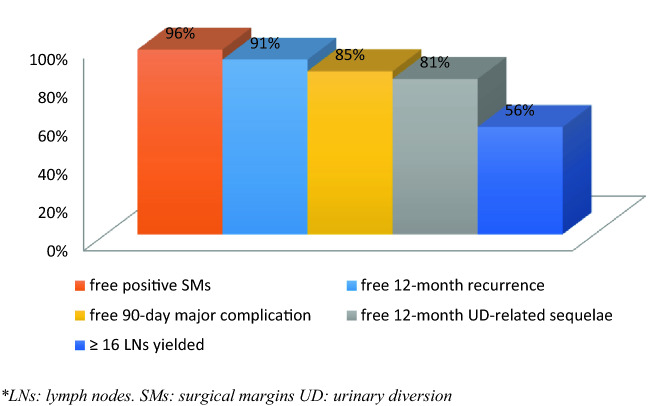
Percentage of RC-pentafecta criteria

A total of 39.4% (*n* = 41) of the procedures validated five criteria of the RC-pentafecta. The percentages of validation of the different criteria of the RC-pentafecta are summarized in Fig. 1.

**Fig. 2:**
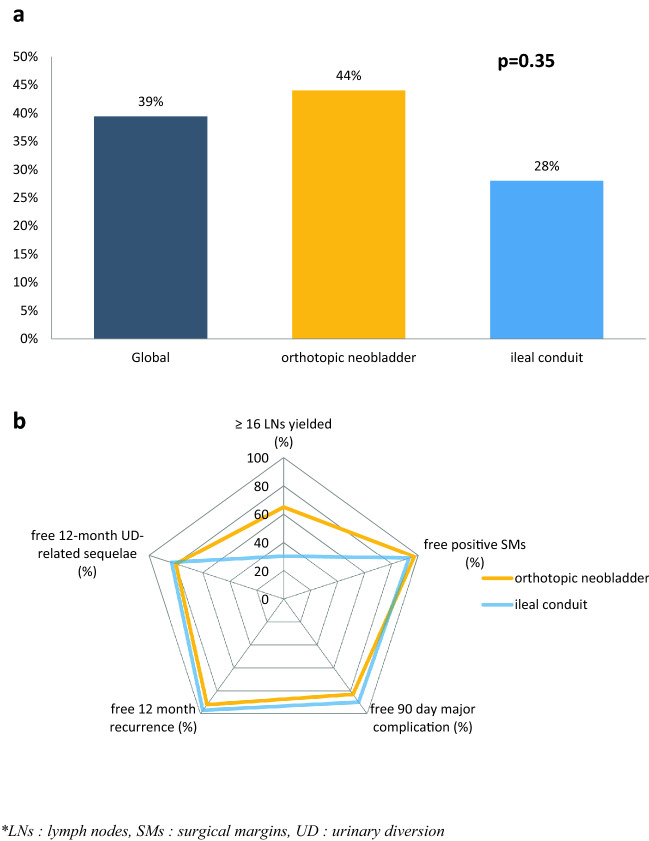
**a** Validation of RC-pentafecta according to the type of urinary diversion. **b** RC-pentafecta criteria according to the type of urinary diversion

The rate of validated RC-pentafecta for orthotopic neobladder and ileal conduit was not significantly different (44.0% vs 27.6%, *p* = 0.37: Fig. 2a). The comparison between orthotopic neobladder and ileal conduit is shown in Fig. 2b.

**Fig. 3:**
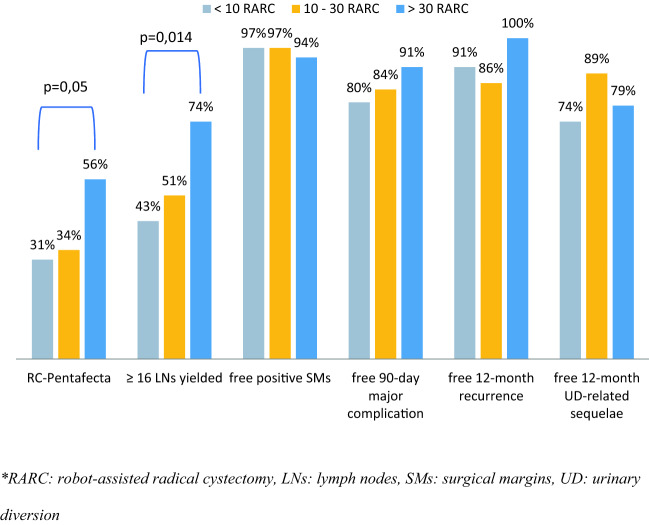
RC-pentafecta validation according to the numbers of procedures

The percentage of patients with validated RC-pentafecta increased with the number of procedures performed by the surgeon; the rate was 31.4% for less than 10 RARC and increased to 55.8% for more than 30 RARC (*p* = 0.05) (Fig. 3).

In univariable analysis, age, presence of diabetes, the readmission rate, and the number of patients with locally advanced disease were significantly different between the "validated RC-pentafecta" and the "non-validated RC-pentafecta" groups (Tables 1, 2).

In multivariable logistic regression analysis, age was the only independent predictor for RC-pentafecta validation (odds ratio [OR] 0.95; 95% confidence interval [CI] 0.90–0.99; *p* = 0.04) (Table 3).

**Table 3:** Multivariate logistic regression analysis

Variables	OR (95% IC)	p
Age	0.95 [0.90; 0.99]	**0.04**
pN + stage	0.31 [0.06; 1.20]	0.11
ASA > 2	0.51 [0.13; 1.68]	0.29
> pT3	0.71 [0.22; 2.13]	0.55
Diabetes	0.96 [0.28; 2.80]	0.99
Type of UD	0.94 [0.29; 3.06]	0.91

Bold values mean statistically significant

^*^ASA, American society of anesthesiologists

^*^UD, urinary diversion

**Fig. 4:**
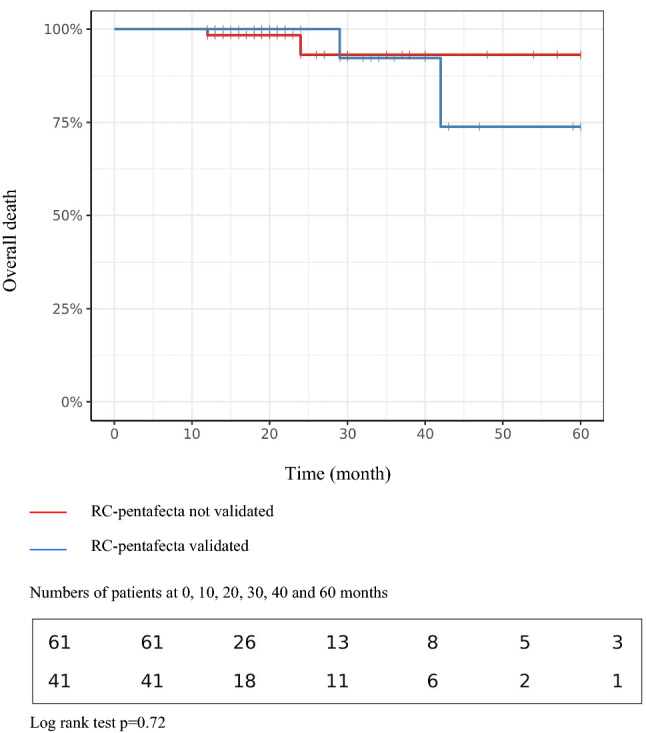
Kaplan–Meier survival curves comparing the groups validated vs not validated RC-pentafecta: overall survival at 5 years

With a median follow-up of 18 months, the 5-year overall survival (OS) rate did not differ significantly between the groups. Indeed, at 60 months, the OS rate was 73.8% in the "validated pentafecta" group vs. 93.2% in the "non-validated pentafecta" group (*p* = 0.78) (Fig. 4).

## Discussion

The current study represents a component of the process of evaluating the quality of surgical procedures and the impact of the learning curve in onco-urology. Our findings confirm that the RC-pentafecta proposed by Cacciamani et al. is reproducible in a European cohort of patients undergoing RARC-ICUD for bladder cancer and that its validation increases with the surgeon’s experience.

In our study, the rate of "validated RC-pentafecta" was 39.4% compared to 53% in the study by Cacciamani et al. The validation of the RC-pentafecta in our cohort was mainly affected by the number of removed lymph nodes and the rate of complications.

PLND has two objectives: prognostic staging and therapeutic. However, its clinical impact remains controversial. The quality of PLND is difficult to assess and remains a source of debate. It has been suggested that OS was correlated with the number of removed lymph nodes [15], and several studies have suggested a minimum number of 10 to 16 nodes [16, 17] as a cut-off. May et al. showed that the removal of 16 lymph nodes improved specific survival by 11% [18].

Several retrospective studies have shown that extensive PLND improves 5-year recurrence-free survival compared to standard dissection [19–23]. A meta-analysis of 19,793 patients confirmed that PLND improved oncological results without being able to demonstrate a difference in the extent of lymph node dissection performed [24]; however, the study presented very heterogeneous data. In addition, a recent prospective study showed no significant difference in OS or specific survival depending on the type of dissection (extensive vs. standard) [25].

The technique for calculating the number of lymph nodes during anatomopathological analysis has not yet been standardized. Indeed, counting the lymph nodes in bloc would increase the total number of lymph nodes found in the dissection, which highlights the influence of the anatomopathological technique used [26]. In our study, the mean number of lymph nodes was 17.8, which is much lower than that reported by Cacciamani et al. (mean 41.3). For less than 10 RARCs, the dissection rate with more than 16 lymph nodes was 43%, which increased to 74% after the 30th RARC. With a dissection rate of ≥ 16 lymph nodes of 56% versus 93% in the study by Cacciamani et al., this factor strongly modifies our results for the validation of pentafecta. Different methods of assessing the number of lymph nodes are likely to be partly responsible for this difference. In the future, to homogenize and improve the evaluation of the quality of surgical excision during cystectomy, the criterion “number of lymph nodes yield” could be modified by a criterion “the extent of PLND”. We found in our study a relative high ratio of pT0 that did not received NAC. This is a bit higher than what is reported in the literature where approximately 10% of patients (6–41% depending on preoperative risk factors) no tumor is found in the specimen [27, 28]. One of the reason of this results might be inclusion criteria that included high-risk NMIBC.

UD-related events account for a major proportion of complications following RC and directly affect the long-term renal function and quality of life of patients [11]. The rate of overall urinary complications in our study was higher than that in the study by Cacciamani et al. (19.2% vs 7.8%). This difference may be explained by the greater number of orthotopic neobladder procedures in our series, which increases the risk of urinary complications, especially leakage in the early postoperative period [11]. The greater number of orthotopic neobladder is probably secondary to a selection bias and a center side effect.

A recent multicenter study try to validate the Cacciamani ‘s RC-pentafecta [29] The authors performed a multicentric external validation of the RC-pentafecta. Among the 730 patients included 208 (28.5%) acheieved the RC-pentafecta. At the multivariate analysis revealed that RC-pentafecta was a significant predictor of overall death. In their multicenter validation, Oh et al*.* considered only the uretero-enteric strictures, arguing that several events overlapped with postoperative complications factors within 90 days and stoma-related complications were shown to only minimally influence survival outcomes. Accordingly to the EAU- ad-hoc Panel For Complication reporting, in contrast to a 90- complication, the sequelae of a procedure should be defined as an after-effect of that procedure [30]. Accordingly, in the RC-Pentafecta proposed by Cacciamani et al*.* the UD-related sequelae are not overlapping with postoperative complications at 90 days [7]. Some of these sequelae may occur months after surgery; therefore, long-term and tightened follow-up of patients with UD is of paramount importance [10] Considering the impact on health-related quality of life of all these long-term UD-related sequelae, regular follow-ups in patients undergoing RC is imperative. Prospective evaluation of complications and side effects of treatments based on patient-reported data seems essential.

The overall recurrence rate was similar to that reported by Cacciamani et al. (15.4% vs 18.5%). Interestingly, there was a significantly higher rate of NAC in our series compared to that observed in the American series (68.2% vs 24.4%). The learning curve was also found to have an oncological impact; in fact, there was no recurrence in the patients operated by experienced surgeon (> 30 RARC), suggesting that this surgery should be referred to expert centers with experienced operators.

Another possible explanation for the differences in the number of the validated RC-pentafecta compared to the original study is that 68.3% of the cystectomies were performed by inexperienced surgeons who had performed less than 30 procedures in our study. Although this ratio likely impacted our validated RC-pentafecta rates, and despite the important difference in terms of surgeon’s experience, our results were not widely different, and, therefore, could not fully explain this difference.

Our series confirms the significant influence of the learning curve, with a net increase in the rate of validated RC-pentafecta from 31% in the early stage of the learning curve to 56% for experienced surgeons. Furthermore, centers in which only one surgeon performed the RARC had the best results.

In multivariable logistic regression analysis, we found age to be the only significant predictor of RC-pentafecta validation; this was likely due to the effect of age on high-grade complications.

In the study of Cacciamani et al., the pentafecta was related to overall survival. However in our study this could not be highlighted. This is might be secondary to the lower number of patients and events, resulting in a lack of statistical power as well as the number of patients who received NAC or who had a high-risk NMIBC at time of surgery.

Our study has several limitations. First, this was a retrospective study, and consequently had some missing data. Second, the multicentric character implies great variability in the surgical technique or the experience of the operators. Third, according to the RC-pentafecta definition all patients with less than 12 months of follow-up were excluded; therefore, the number of included patients was low, which led to a lack of capacity to confirm, in particular, an advantage of the pentafecta on survival and the significance of certain data in the multivariate analysis. We believe that another tool such as the trifecta proposed by Brasseti et al. would had been also interesting to evaluate especially due to less debatable criteria. More studies are awaited to decide which tool should be used and which criteria are of utmost importance in the future.

## Conclusion

Our study confirms that the RC-pentafecta proposed by Cacciamani et al. is reproducible in a European cohort of patients undergoing RARC-ICUD for bladder cancer and that its validation increased with the surgeon's experience. It appears to be essential to change the evaluation of surgical procedures from purely quantitative criteria to qualitative composite criteria such as the one proposed by the pentafecta.
